# Biomarkers of disease progression in progressive supranuclear palsy for use in clinical trials

**DOI:** 10.1093/braincomms/fcaf022

**Published:** 2025-01-16

**Authors:** Cassandra Marotta, Benjamin Sinclair, Terence J O’Brien, Lucy Vivash

**Affiliations:** Department of Neuroscience, School of Translational Medicine, Monash University, Melbourne, VIC 3004, Australia; Department of Neuroscience, School of Translational Medicine, Monash University, Melbourne, VIC 3004, Australia; Department of Neuroscience, School of Translational Medicine, Monash University, Melbourne, VIC 3004, Australia; Department of Neurology, Alfred Health, Melbourne, VIC 3004, Australia; Department of Neuroscience, School of Translational Medicine, Monash University, Melbourne, VIC 3004, Australia; Department of Neurology, Alfred Health, Melbourne, VIC 3004, Australia

**Keywords:** progressive supranuclear palsy, biomarkers, disease progression, clinical trial, sample size

## Abstract

Progressive supranuclear palsy (PSP) is a rare neurodegenerative disease with no current disease-modifying treatments approved. Longitudinal research and clinical trials for PSP are ongoing and require reliable measures that are sensitive to disease progression. Despite susceptibility to subjective limitations, clinical and cognitive assessments are the most used instruments in therapeutic trials in PSP. The objective of this review was to identify measures that have been studied longitudinally as measures of progression and are suitable for use as clinical trial endpoints. We reviewed the measures currently used as trial endpoints, identifying the clinical, cognitive, fluid and imaging measures that have previously been studied longitudinally, and discuss current diagnostic and emerging measures that are yet to be studied longitudinally but that may be sensitive to disease progression. We found that many fluid and imaging measures require further research to validate their use as longitudinal measures of change, including emerging measures that have not yet been studied specifically in PSP. We also summarize the sample size estimates required to detect changes in a two-arm, 52-week therapeutic trial and found that specific MRI volumes require the smallest sample sizes to detect change.

## Teaser

In this review, Marotta *et al*. provide a summary of all clinical, cognitive, biofluid and imaging measures that have been studied longitudinally and in clinical trials in progressive supranuclear palsy (PSP). The authors also highlight emerging measures that have the potential to become future biomarkers of disease progression in PSP.

## Graphical Abstract

**Graphical abstract:**
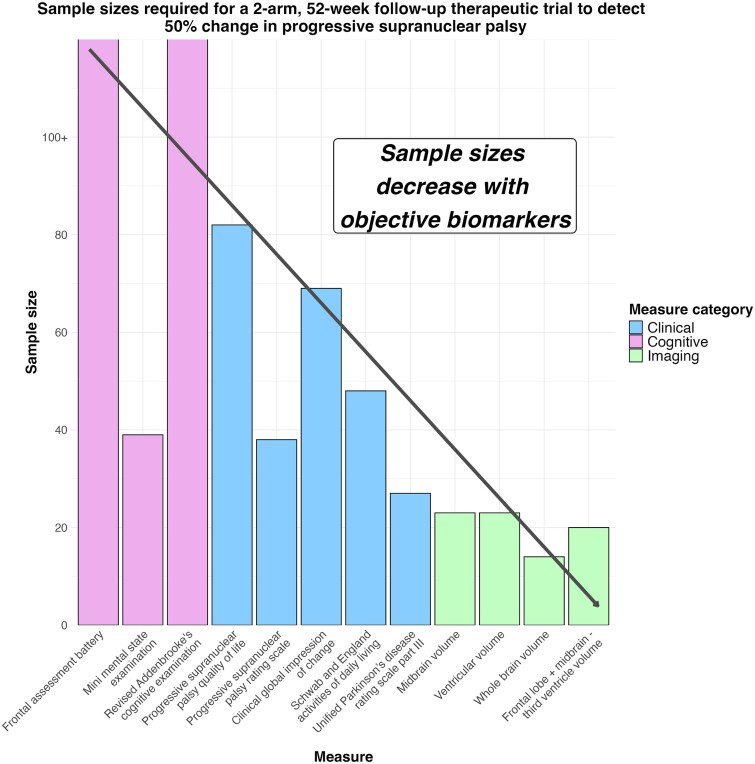

## Introduction

Progressive supranuclear palsy (PSP) is a rare, devastating, relentlessly progressive neurodegenerative movement disorder with a prevalence of ∼6 per 100 000.^1-4^ Pathologically, PSP is characterized as a ‘tauopathy’ due to the accumulation of the hyperphosphorylated protein tau within the brain, with particularly severe tau aggregation in the basal ganglia, dentate nucleus of the cerebellum and the parietal and frontal lobes.^5^ PSP is diagnosed primarily on the basis of clinical features, with no currently available biomarkers to aid diagnosis. Moreover, PSP is typically diagnosed only after more common diseases, such as Parkinson's disease, have been excluded. A variety of PSP subtypes have been described all with differences in their predominant symptoms, including progressive gait freezing, parkinsonism, frontal presentation, ocular motor dysfunction, speech/language disorder, corticobasal syndrome, postural instability, primary lateral sclerosis and cerebellar ataxia. The most common subtype is PSP Richardson's syndrome (PSP-RS) which has a survival rate of ∼6–7 years from symptom onset and is characterized by the early onset of postural instability, falls and vertical saccadic dysfunction.^1-4,6,7^

### The need for biomarkers of disease progression

There are no effective disease-modifying treatments that target the underlying pathology of PSP, with the current treatments consisting of physiotherapy, occupational therapy and symptomatic pharmacological treatment (typically anti-parkinsonian dopaminergic treatments or anti-depressants, anxiolytics and anti-psychotics).^8^ There is an urgent unmet need for the development of new biomarkers for diagnosis, prognosis and endpoints in clinical trials of new therapies, in particular the development of disease-modifying therapies to halt the progression of PSP. The US Food and Drug Administration (FDA) requires that primary efficacy endpoints should be clinically meaningful measures of disability or surrogate markers (e.g. imaging or biofluid markers) which indirectly measure and predict a clinical benefit. However, in rare neurodegenerative diseases such as PSP, identifying appropriate surrogate endpoints is challenging, and therefore, clinical endpoints are relied upon. Increasingly, the FDA is encouraging patient-centred outcome measures, involving patients in the identification of important outcomes, as well as the development of tools sensitive to the detection of changes in such measures and subsequent incorporation of these measures into regulatory trials.^9^

The PSP rating scale (PSPRS), a clinical scale of severity, is the most widely used endpoint measure in trials despite several limitations, including variability in the administering clinician's expertise, subjective scoring rubrics and the influence of practice, observer and treatment effects.^10^ Objective, quantitative biomarkers of disease progression are lacking in PSP but are needed to corroborate clinical scales and to accurately evaluate treatment efficacy in clinical trials. Research is needed to identify biomarkers that may serve as suitable surrogate endpoints that accurately reflect and predict clinical benefit in clinical trials. The aim of this review was to identify biomarkers (including clinical measures) that are capable of tracking disease progression in PSP and are suitable for use as clinical trial endpoints.

A literature search was conducted in PubMed for the years 2000–2023. The search terms used were (‘Progressive Supranuclear Palsy’ OR ‘PSP’) AND (‘survival’ OR ‘longitudinal’ OR ‘progression’ OR ‘follow up’), which resulted in 267 results. Papers were included in this review if they longitudinally studied progression tracking measures where a measure was assessed at baseline and follow-up visits. Papers were excluded if they were not in English, were review articles or case studies, did not have an independent PSP group, reported only on survival rates or were focussed on biomarkers for diagnostic purposes only. Forty papers from the PubMed search and additional papers based on a review of reference lists and manual selection were included in this review. Additionally, a search of ClinicalTrials.gov, Australian New Zealand and European Union Clinical Trials registries was also conducted to identify past and ongoing clinical trials of therapeutic drug interventions in PSP. Studies investigating new diagnostic techniques were excluded. Of the search results, 29 interventional drug trials reported endpoints aimed at evaluating treatment efficacy.

## Existing measures

### Clinical measures

There are many clinical assessments which can measure disease progression longitudinally that have been used in PSP. These include both general clinical assessments and measures which have been developed specifically for PSP.

#### PSP Rating Scale

The PSPRS is a clinical scale of symptom severity specific to PSP, consisting of 28 questions scored on a Likert scale, giving a total score of 0–100.^10^ Table 1 reports the annual PSPRS decline from a number of observational studies and clinical trial placebo arms with 12-month follow-up assessment. All studies report on diagnosis at baseline with a mean disease duration ranging from 1.4 to 4.0 years in studies reporting only subjects with PSP-RS and 3.1 to 4.5 in studies where PSP subtype was not specified. In addition, Street *et al.*^11^ also report results based on final diagnosis which accounts for additional information obtained throughout the study. The final diagnosis exhibited smaller changes in PSPRS scores compared with the intention to treat (9.9 compared with 11.6 points for the PSP-RS subtype).^11^ The same study also reports results for both individual PSP subtypes as well as a PSP total cohort and a number of supplementary tables which present data by diagnosis timepoint and subtype for a large number of measures and biomarkers. Across all studies, the PSP-RS subjects mean PSPRS score at baseline ranged from 29.7 to 40.4 points and the annual progression rate ranged from 8.7 to 11.6 points, while for the unspecified subtype studies, the mean baseline score ranged from 32.1 to 42.0 points and the annual progression rate ranged from 8.1 to 14.8 points (Table 1, Supplementary Table 1).^10-26^

**Table 1:** Mean annualized change in score on the PSPRS

Study	Sample size	Mean disease duration	Mean baseline PSPRS	Mean annual PSPRS decline
Golbe^10^	65	3.8	42.0	9.7
**Ghosh** ^13^	**16**	**4.0**	**32.8**	**11.3**
Josephs^15^	28	3.1	36.4	14.7
Litvan^14^	27	3.1	33.5	9.1
Street^12^	109	3.4	34.7	8.1
**Street** ^12^	**80**	**3.1**	**-**	**8.7**
Street^11^	99	-	-	9.9
**Street** ^11^	**52**	**-**	**-**	**11.6**
Street^11a^	117	4.5	33.0	9.0
**Street** ^11 a^	**57**	**3.5**	**31.3**	**9.7**
**Grötsch** ^16 b,c^	**86**	**3.0**	**-**	**11.1**
**Bang** ^17 d^	**241**	**-**	**39.1**	**11.1**
Höglinger^18c,d^	99	-	37.8	9.5
**Tsai** ^19 d^	**198**	**-**	**38.9**	**9.6**
**Quattrone** ^20 c,d,e^	**105**	**1.4**	**38.0**	**10.0**
***Boxer*** ^21^ *^d^*	***123***	***-***	***39.0***	***10.9***
***Tolosa*** ^22^ *^c^*	***21***	***3.04***	***40.4***	***10.4***
*Leclair-Visonneau* ^23^	*14*	*3.6*	*32.1*	*14.8*
*Apetauerova* ^24^	*16*	*-*	*39.0*	*11.8*
***Nuebling*** ^25^	***14***	***-***	***29.7***	***10.8***
***Dam*** ^26^ *^e^*	***138***	***1.8***	***37.3***	***10.6***

Papers in bold font are based on only PSP-RS subjects. Papers in italicized font are clinical trials results.

^a^Based on final diagnosis.

^b^Placebo and treatment groups combined.

^c^Includes subjects from NCT01049399.

^d^Includes subjects from NCT01110720.

^e^Includes subjects from NCT03068468.

However, when studying participants with shorter disease duration (mean 1.9 years), Pereira *et al.*^27^ found that the change in PSPRS score exhibited slower progression of 0.6 points per month (∼7.2 points per year). Golbe *et al.*^10^ also found PSPRS progression rates were slower in patients earlier visits (8.7 points per year) than at third follow-up visits more than 6 months after the second (12.4 points per year).

Of the 29 therapeutic drug trials evaluating efficacy that were identified in the clinical trial registries search, 15 trials, including one long-term extension study, have listed the PSPRS as the primary endpoint. Of the remaining 14 trials, the primary endpoint in 10 was safety and tolerability measures, and in nine, the PSPRS was included as a secondary endpoint.

Of the six completed clinical trials using the PSPRS as the primary endpoint, five have reported results. In these trials, the mean PSPRS score at baseline ranged from 32.1 to 40.4 points, and the annual change ranged from 9.9 to 14.8 in the placebo groups, with similar annual rates of change in the treatment groups (Table 1).^21-24,26^

#### PSPRS subdomains

Prediction of disease progression in individual subdomains using baseline total PSPRS score showed that the Gait/Midline subscore exhibited the fastest deterioration over a 1-year period.^28^ In a study of 27 subjects (mean disease duration of 3.17 years), Litvan and Kong^14^ determined that to detect a 50% reduction in the rate of progression in the Ocular Motor subdomain, a two-arm, 1-year follow-up trial would require only 25 subjects per arm and 97 per arm for the Gait/Midline subdomain (Supplementary Table 2). However, another study with a much larger sample size (*n* = 187) estimated the Ocular Motor subdomain would require 109 subjects, while the Gait/Midline subdomain required the smallest sample size of all subdomains with 63 subjects per arm, which was reduced further to 53 subjects per arm when excluding people with a disease duration over 5 years.^29^

#### Modified PSPRS

Grötsch *et al.*^16^ developed a condensed version of the PSPRS, the modified PSPRS (mPSPRS) through the removal of several items following consultation with 16 PSP experts. The aim was to improve the PSPRS by removing items that were not sensitive measures of change over typical trial durations and that pertained to clinical features already covered by other items. Fourteen of the original PSPRS items were kept, and the total score ranged from 0 to 28.^16^ The mPSPRS can detect change over 6 and 12 months, with the annual rate of progression ranging from 3.2 to 4.8 points (Supplementary Table 2).^11,16^ To detect a 50% change in progression rate, 41 patients per arm would be needed in a two-arm, 1-year follow-up trial (Supplementary Table 2).^16^

#### PSPRS-10

The FDA has also recommended a condensed version of the PSPRS for use in clinical trials which contains 10 of the original PSPRS items. This scale adheres to the FDA guidelines for patient-focussed outcome measures, although clinical trial sponsors should seek specific approval from regulators prior to use as a trial endpoint. Furthermore, the FDA also recommended a rescored version (rPSPRS-10) where response categories were combined in nine of the test items. Gewily *et al.*^30^ used item response theory (IRT) to investigate how the items on each of the three PSPRS versions compared in their discriminative power and ability to measure a subjects disability and measure longitudinal disease progression. The IRT analysis showed that the full-length PSPRS lacks the unidimensionality that is expected from a scale that accurately measures disease severity. Of the original 28 PSPRS items, the 10 items from the PSPRS-10 were the most informative of disease severity. The PSPRS-10 performed better than the rPSPRS-10 and PSPRS, with greater pairwise correlations between test items and smaller sample sizes required to detect a 50% change in a two-arm RCT (*n* = 38 in the PSPRS-10 compared with 102 for the full-length PSPRS) (Supplementary Table 2).^30^

#### Unified Parkinson's Disease Rating Scale

The Unified Parkinson's Disease Rating Scale (UPDRS) was designed to measure severity of impairment in Parkinson's disease (PD).^31^ It is also used in studies of PSP, possibly because it is a familiar scale among movement disorder clinicians; however, core features of PSP such as ocular motor dysfunction are insufficiently evaluated. In particular, the motor section (Part III) of the UPDRS is frequently used to measure disease severity in PSP. In PSP, the UPDRS (Part III) mean annualized change in score ranges from 8.3 to 11.75 points (Supplementary Table 1).^11,13,27^ Litvan and Kong^14^ found that the UPDRS total score progressed annually by 11.4 points. The UPDRS has been used as the primary endpoint in one PSP clinical trial (NCT00382824) where the mean annual change in the placebo group was 12.8 points and 11.5 points for the treatment group (Supplementary Table 1).^24^

#### Schwab and England Activities of Daily Living Scale

The Schwab and England Activities of Daily Living Scale (SEADL) is an assessment to measure activities of daily living, originally designed for PD.^32,33^ Although it is not specific to PSP, it has an annualized progression rate of −11.1 to −17.1% and greater impairment at baseline assessment is a strong predictor of subsequent annual change on the PSPRS (Supplementary Table 1).^11,14,17,18,20^ The SEADL has been used as the co-primary endpoint along with the PSPRS in one therapeutic intervention clinical trial where the annual rate of decline was reported as 17 and 16% in the placebo and treatment groups.^21^

Other clinical measures that have been longitudinally investigated in PSP are outlined in Supplementary Table 1. The PSP Quality of Life (PSP-QoL) is a 45-item, patient-reported scale designed to include specific features of PSP such as ocular motor disturbances.^34^ Although the PSP-QoL is a specific measure in PSP, it is still susceptible to limitations such as its length and confronting nature for patients. A shortened, 12-item version of the PSP-QoL, the PSP-ShoQoL has recently been developed to reduce patient burden.^35^ The PSP-ShoQoL significantly correlates with the PSP-QoL, exhibits an annual score increase of 2.46 points and has a similar effect size (−0.25) to the PSP-QoL (−0.27).^11,35^ Other clinical scales studied include the Clinical Global Impression of Change (CGIC), which assesses global function before and after commencing a treatment.^36^ It had a strong correlation and effect size equal to that of the PSPRS, larger than other clinical scales investigated when measuring annual change,^17^ and is included as a secondary endpoint in 10 clinical trials. The Geriatric Depression Scale (GDS), used for assessing depression in older adults, is also used as a secondary endpoint in three trials.^17^ However, generic assessments such as the CGIC and the GDS are broad scales that are measures of general well-being and change rather than specific symptoms of PSP. They are not sensitive to many prominent features of PSP and may not reflect specific disease progression or mitigation.

### Cognitive measures

Cognitive measures have been studied longitudinally to assess their ability to measure progression in PSP. However, cognitive measures are also susceptible to a number of limitations when administering the assessments. In PSP, participants could have difficulty completing tests due to other disease symptoms such as gaze palsy, neck rigidity, communication issues and the inability to hold a pencil, rather than actual cognitive deficits.

#### Frontal Assessment Battery

The Frontal Assessment Battery (FAB) is a 10-min assessment designed to evaluate executive function.^37^ It has been used as a primary endpoint in one clinical trial (NCT04184063) and as a secondary endpoint in four PSP clinical trials; however, to date, no results have been reported. In PSP, FAB performance has been found to decline by 3.8 points over 2 years;^15^ however, another two studies found no significant change over 1 year (Supplementary Table 1).^13,14^

#### Repeatable Battery for Assessing Neuropsychological Status

The Repeatable Battery for Assessing Neuropsychological Status (RBANS) was developed to evaluate abnormal cognitive decline in older adults by assessing five domains: immediate memory, delayed memory, visuospatial/constructional function, attention and language.^38^ In a study by Duff *et al.*,^39^ all five indices exhibited a significant decline 6 months after baseline assessment, although at 12 months this had stabilized in most indices and decline was only observed in the visuospatial/constructional and attention indices.

Phonemic fluency has been included as a secondary endpoint in two completed clinical trials; however, neither found significant annual changes (Supplementary Table 1).^22,26^ A study by Fiorenzato *et al.*^40^ also found that there was no significant change in this measure over 15 months. In contrast, significant impairment was observed on a number of other cognitive assessments at 15-month follow-up, including Semantic Fluency, Digit Span Sequencing, Benton's Judgment of Line Orientation test and the Montreal Cognitive Assessment.^40^ There have also been a number of cognitive measures studied longitudinally that have shown inconsistent results regarding their sensitivity to measure disease progression. Ghosh *et al.*^13^ found no annual changes on the Hayling test or Revised Addenbrooke's Cognitive Examination (ACE-R) (Supplementary Table 1). However, Street *et al.*^12^ found that ACE-R progression was significantly faster in subjects with PSP-RS than in subjects with other PSP variants (∼−5.3 and −3 points per year, respectively). The Mini Mental State Examination is used in the inclusion criteria in 14 of the 29 clinical trials; however, while Litvan and Kong^14^ found annual worsening of −2.1 points, other studies have reported no significant changes for up to 24 months (Supplementary Table 1).^27,40^

### Fluid measures

Cerebrospinal fluid (CSF) and blood measures have strong potential as diagnostic and disease progression biomarkers as they are objective and not susceptible to the subjective influences that can affect clinical and cognitive measures. However, fluid biomarkers are often not disease-specific, reflecting broad neurodegeneration, and very few studies have investigated these measures longitudinally. Currently, fluid measures are not used as primary endpoints in clinical trials, although they have been included as secondary endpoints (Supplementary Table 1).

#### Tau markers

When compared with healthy controls, concentrations of CSF total tau (t-tau) and phosphorylated tau (p-tau)181 tend to be normal or low in PSP.^41-43^ This may be because the predominant tau isoform in PSP, four-repeat (4R) tau, is not released from cells as freely as three-repeat tau and therefore exhibits lower levels in CSF.^44^ Similar patterns are seen in a number of neurodegenerative disorders, particularly in atypical parkinsonian syndromes (APS).^41,42,45^ Magdalinou *et al.*^45^ found no significant differences in t-tau and p-tau181 levels when comparing PSP with healthy controls and APS. Hall *et al.*^42^ found that p-tau181 levels in PSP were 12% lower than in healthy controls, but there was no significant difference when compared with PD or multiple system atrophy subjects. T-tau levels in PSP (median 429 ng/mL) were significantly different from Alzheimer's disease subjects (median 840 ng/mL) but not healthy controls, PD or multiple system atrophy subjects (median levels of 473, 371 and 528 ng/mL, respectively).^42^ When studied longitudinally in PSP participants, Boxer *et al.*^46^ and Bäckström *et al.*^47^ found no changes in either CSF p-tau181 or t-tau over a 1-year period (Supplementary Table 1). To date, t-tau and p-tau181 have not been studied in blood longitudinally in patients with PSP.

#### Neurofilament light chain

Neurofilament light chain (NfL), which reflects non-specific axonal damage, exhibits higher concentration levels in PSP than both healthy controls and PD subjects.^42^ Magdalinou *et al.*^45^ reported that NfL levels could differentiate PSP (median levels of 2219 ng/L) from healthy controls (560 ng/L), PD (966 ng/L) and multiple system atrophy (3024 ng/L). A number of studies have found significant increases in CSF NfL levels over time in patients with PSP studied serially.^45-48^ Bäckström *et al.*^47^ found that in PSP subjects with a short disease duration (13 months median) CSF NfL increased by 27.1% over 12 months, while it remained unchanged in PD and multiple system atrophy subjects. Boxer *et al.*^21^ found that annual increases in CSF NfL correlated with changes on the PSPRS ocular motor subscore and superior cerebellar peduncle (SCP) volume.

Furthermore, ratios of biofluid markers can also discriminate PSP from healthy controls with higher levels of Glial Fibrillary Acidic Protein (GFAP)/t-tau and NfL/t-tau in CSF and plasma and lower levels of GFAP/NfL in CSF.^49^

### Neuroimaging measures

Visual inspection of neuroimaging can be used to support a clinical diagnosis of PSP;^6^ however, to date no quantitative imaging biomarkers have been utilized for clinical diagnosis or study inclusion criteria. There have been many studies which have investigated the quantitative measure of midbrain atrophy and other neuroimaging findings as potential biomarkers of both diagnosis and progression.

#### Midbrain volume

In 2006, Paviour *et al.*^50^ calculated that in PSP subjects with a mean disease duration of 4.6 years, the mean annualized rate of midbrain atrophy was 2.2% which was greater than the atrophy rate of the whole brain (1.2%) (Supplementary Table 1). A clinical trial^51^ evaluating a treatment that resulted in a 40% reduction in the rate of atrophy would require only *n* = 82 subjects compared with other brain regions which would require much larger groups (*n* > 170; Supplementary Table 2). Bang *et al.*^17^ found that midbrain atrophy had the largest annual effect size of MRI measurements. The rate of midbrain atrophy over both 6 and 12 months (1.25–3.5%) correlates with progression on the PSPRS in a number of studies^17,19,52,53^ and the sample size required to detect a 50% reduction in midbrain atrophy rate in a two-arm, 1-year follow-up trial could be as small 23 patients per arm (Supplementary Table 2).^53^

#### Whole-brain volume

Although not specific to PSP, whole-brain atrophy has been studied as a measure of progression in PSP in a number of longitudinal studies. Whole-brain atrophy rates correlate with poorer performance on a number of clinical and cognitive scales, including the PSPRS^19,51^ although Guevara *et al.*^54^ found no correlation between annualized change in clinical measures and whole-brain volume change. Sample size estimates for detecting a reduction in annual whole-brain atrophy rate in a two-arm clinical trial range from 14 to 225 patients per arm so caution should be taken when using it to measure disease-specific progression (Supplementary Table 2).^18,19,51,54^

The volumes of many other brain regions have also been investigated to measure progression in PSP. The Magnetic Resonance Parkinsonism Index (MRPI 2.0) which is an extension of the MRPI (pons/midbrain area ratio multiplied by middle cerebellar peduncle width/SCP width ratio) calculated by multiplying the MRPI by third ventricle width exhibits an annual change of 15.0–17.69%.^20,55^ Quattrone *et al.*^20^ found that of the imaging measures studied, the MRPI 2.0 exhibited one of the highest annualized percentage changes (10–15%) and it also exhibited high variability between subjects. Ventricular volume has an annualized volume expansion of 11.8% compared with 4.19% in healthy older adults (Supplementary Table 1).^11,56^ Ventricular volume change has one of the largest effect sizes for annual change of all MRI measures and correlates with progression on the PSPRS.^17^ Frontal lobe volume also exhibits one of the fastest regional rates of decline (1.84–4.0%) (Supplementary Table 1). Combining several regions that are known to be affected in PSP further reduces the sample size required to detect possible treatment effects. Höglinger *et al.*^18^ determined that a volume composite consisting of the frontal lobe, midbrain and third ventricle required the smallest sample size of 20 patients per arm to detect a 50% reduction in atrophy in a two-arm, 1-year clinical trial (Supplementary Table 2). It has also been shown to outperform PSP staging systems, including an MRI system sequencing the progression of brain atrophy across different regions and neuropathological systems reflecting the sequence of tau accumulation.^20^ This volume composite is currently the only neuroimaging measure to be used as the primary endpoint in a clinical trial.^57^

#### Diffusion-weighted MRI

**Figure 1:**
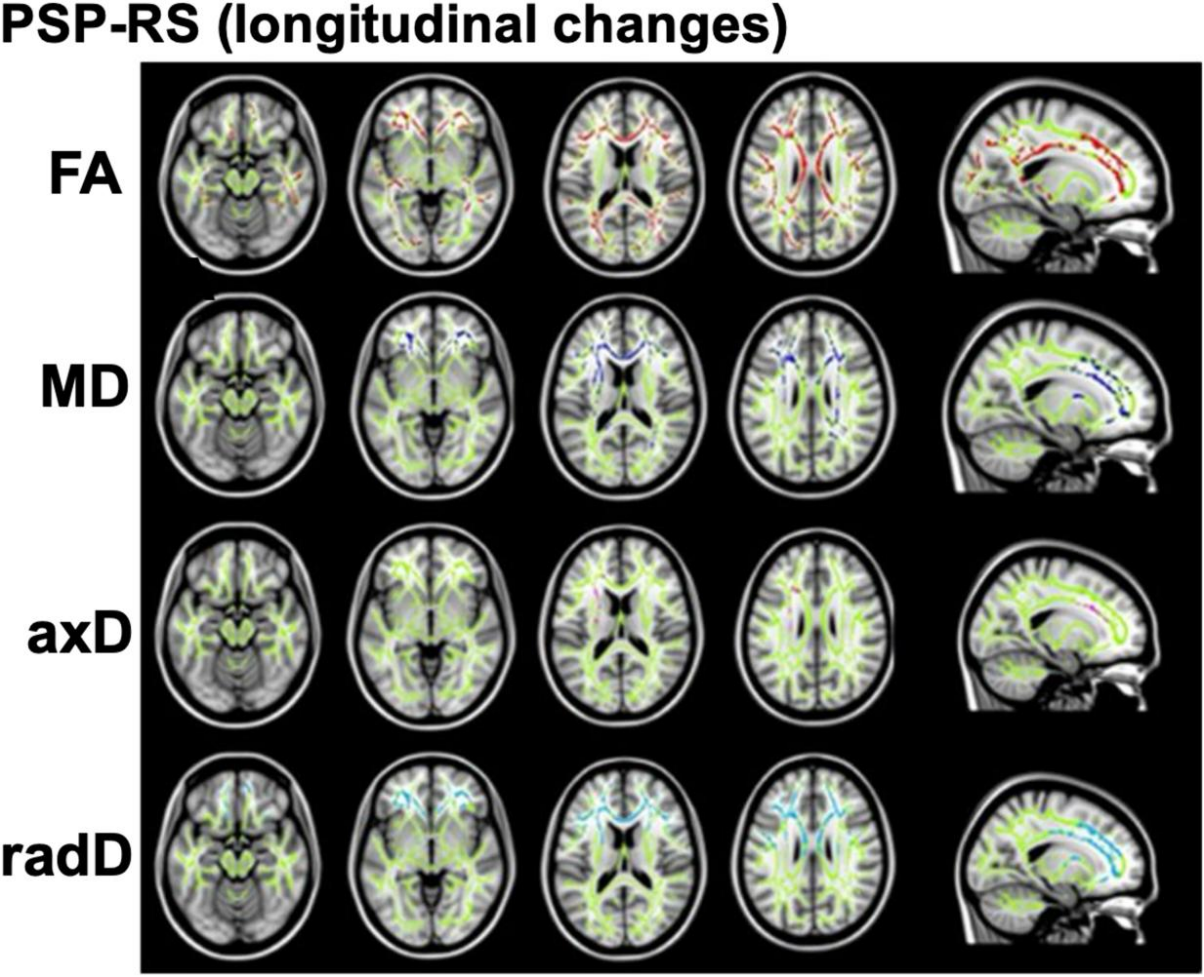
**Longitudinal white matter changes in DTI tract-based spatial statistics in progressive supranuclear palsy Richardson’s syndrome. Decreased FA (red) and increased MD (blue), axial diffusivity (axD) (pink) and radial diffusivity (radD) (cyan) projected onto a white matter skeleton (green)**. *n* = 21, *P* < 0.05, threshold-free cluster enhancement corrected. Columns 1–4 display axial slices of the brain, increasing superiorly. Column 5 displays a sagittal slice of the left hemisphere. PSP-RS, progressive supranuclear palsy Richardson's syndrome. Figure from Agosta *et al*.^59^

Diffusion-weighted imaging has been used to longitudinally study white matter damage and microscopic changes in PSP. The apparent diffusion coefficient shows that PSP subjects have longitudinal increases in the putamen greater than that exhibited in healthy controls.^58^ A diffusion tensor imaging (DTI) study^59^ showed increased mean diffusivity (MD) and radial diffusivity and decreased fractional anisotropy (FA) in the corpus callosum, frontoparietotemporal tracts and anterior thalamic radiations after a mean of 1.4 years (Fig. 1). Another study by Zhang *et al.*^60^ found that after 6 months, PSP subjects exhibited 3% greater reductions in FA in the SCP compared with healthy controls. Fixel-based analysis (FBA) and free-water (FW) imaging both revealed widespread changes in fibre density, FW and FW-corrected anisotropy over 1 year.^61^ Neurite orientation dispersion and density imaging (NODDI) did not exhibit any significant longitudinal changes in white matter tracts but did show that isotropic volume fraction in the substantia nigra and red nucleus increased greater than in healthy controls over 1 year, indicating an increase in extracellular fluid associated with neurodegeneration. Longitudinal changes exhibited on FBA, FW and NODDI were associated with clinical severity changes when PSP, PD and multiple system atrophy groups were combined.^61^ How these microstructural changes reflect clinical disease severity specifically in PSP is yet to be explored.

#### Positron emission tomography

While midbrain hypometabolism on ^18^F-fluorodeoxyglucose PET may be used as a supportive feature for PSP diagnosis, no longitudinal studies have investigated this as a measure of disease progression. However, numerous clinical trials are underway evaluating treatments that target tau accumulation; thus, tau-binding PET may be useful as a trial endpoint and as a demonstration of proof of mechanism.

Pavone *et al.*^62^ found that greater midbrain ^18^F-flortaucipir standardized uptake value ratios (SUVR) at baseline correlated with PSPRS progression. Greater precentral cortex and supplementary motor area SUVRs correlated with a greater rate of progression on the UPDRS (Part III). Malpetti *et al.*^63^ found that greater ^18^F-flortaucipir binding potential in the midbrain, pons, thalamus, dentate nucleus and cerebellar white matter correlated with faster progression on the PSPRS. The same study also investigated neuroinflammation using ^11^C-PK11195 which primarily binds to activated microglia demonstrating binding potential in the brainstem, dentate nucleus and cerebellar white matter and correlated with faster progression on the PSPRS.^63^ However, these studies did not repeat the PET scans at follow-up to investigate changes over time. An earlier study by Whitwell *et al.*^52^ did investigate ^18^F-flortaucipir SUVR longitudinally. No significant correlations were found between baseline ^18^F-flortaucipir SUVR and subsequent change in SUVR and the regional changes at 12-month follow-up varied across subjects. There was also no correlation between changes in the PSPRS and longitudinal ^18^F-flortaucipir changes in the midbrain, precentral cortex and dentate nucleus.^52^ Thus, based on the few studies available, it appears that unlike CSF tau markers, tau PET imaging shows increases in tau, localized to particular areas. However, quantitative markers to track longitudinal disease progression are yet to be established.

## Emerging biomarkers

The paucity of biomarkers that are both reliable and specific for use as endpoints in trials in PSP means work continues to evaluate biomarkers that are sensitive, specific and reliable for use as trial endpoints. These include biomarkers that have currently been identified for their diagnostic potential in PSP but have yet to be evaluated longitudinally and others which have been studied in other neurodegenerative diseases which could be appropriate as measures of disease progression in PSP. Biomarkers that have yet to be fully tested in PSP but have the potential to be used as measures of disease progression are discussed here.

### Fluid biomarkers

#### Tau and NfL chain

Low levels of CSF p-tau181 and a higher NfL/p-tau ratio predict progression on the PSPRS at 52-week follow-up.^64^ Low SCP volume correlates with higher CSF NfL levels^64^ and higher plasma NfL levels (>37.3 ng/mL) predicted increased severity on the PSPRS, SEADL and RBANS at follow-up.^64^ Higher plasma NfL levels are also predictive of greater whole-brain and SCP volume loss.^65^ However, no association between baseline plasma NfL levels and longitudinal cognitive decline measured by the ACE-R has been found.^66^

#### Brain-derived tau

An assay that selectively binds to brain-derived tau (BD-tau) while avoiding tau from peripheral sources has been investigated for its diagnostic potential in Alzheimer's disease.^67^ BD-tau in serum significantly correlated with paired CSF BD-tau and was able to differentiate Alzheimer's disease from healthy controls with mean levels of 32.4 pg/mL compared with 3.6 pg/mL. Alzheimer's disease could also be differentiated from other non-Alzheimer's disease neurodegenerative diseases, including PSP, primary progressive aphasia, behavioural variant frontotemporal dementia (bvFTD) and corticobasal syndrome. As a combined cohort, the non-Alzheimer's disease neurodegenerative diseases had a mean serum BD-tau level of 4.2 pg/mL.

#### Phosphorylated-tau assays

While p-tau181 is a well-established biomarker in Alzheimer's disease and other neurodegenerative diseases, tau isoforms phosphorylated at alternative sites, such as p-tau217 and p-tau231, are also being investigated as biomarkers of neurodegenerative diseases. Plasma p-tau217 levels are elevated in Alzheimer's disease (mean 0.72 pg/mL) and can differentiate it from healthy controls (mean 0.12 pg/mL) and other neurodegenerative diseases including PSP (mean 0.19 pg/mL). However, there are no significant differences between levels in PSP and healthy controls or other neurodegenerative diseases.^68^ Plasma p-tau231 can also distinguish Alzheimer's disease from non-Alzheimer's disease dementias including PSP and other tauopathies (mean 34.04 pg/mL compared with 28.31 pg/mL); however, no significant differences were seen between any of the non-Alzheimer's disease diagnoses.^69^ CSF p-tau202 and p-tau205 have also been investigated in Alzheimer's disease and found to increase at a later disease stage than p-tau181 and p-tau217, when symptom onset occurs, although these markers are yet to be investigated in PSP.^70^

### Imaging biomarkers

#### Neuromelanin-sensitive MRI

Neuromelanin-sensitive MRI allows for imaging of dopaminergic neurons via the neuromelanin pigment contained within them. A reduction in the neuromelanin volume of the substantia nigra pars compacta was found in PSP where subjects had significantly lower mean volume and signal-to-noise (SNR) ratio (144.8 mm^3^ and 108.0, respectively) than healthy controls (307.1 mm^3^ and 110.1, respectively), PD (188.3 mm^3^ and 110.4, respectively) and multiple system atrophy (186.7 mm^3^ and 109.5, respectively) subjects. When investigating the regional neuromelanin changes those exhibited in PSP were different from the other disorders where the SNR was lower in the associative territory than the sensorimotor and limbic territories in PSP, while the opposite pattern was seen in healthy controls, PD and multiple system atrophy subjects.^71^ Further research is needed to investigate how neuromelanin changes longitudinally.

#### Quantitative susceptibility mapping

Quantitative susceptibility mapping (QSM) allows for the quantification of iron content using magnetic susceptibility.^72^ Increased iron is associated with microglial activation, demyelination and protein accumulation, including of hyperphosphorylated tau.^73^ Increased susceptibility values in the red nucleus have been shown to differentiate PSP from PD and healthy controls.^74,75^ Combining the susceptibility values of the red nucleus with NfL levels further improved the diagnostic accuracy.^75^ Mean susceptibility values are higher in PSP patients than PD and healthy controls and significantly correlate with disease severity on the PSPRS, particularly in the substantia nigra and red nucleus.^76^ More research is needed to determine whether excessive iron accumulation is an initial trigger of, increases the rate of or is a result of degeneration.^73^ Longitudinal studies are also needed to determine whether QSM can be used as a measure of disease progression.

#### Second-generation tau PET

**Figure 2:**
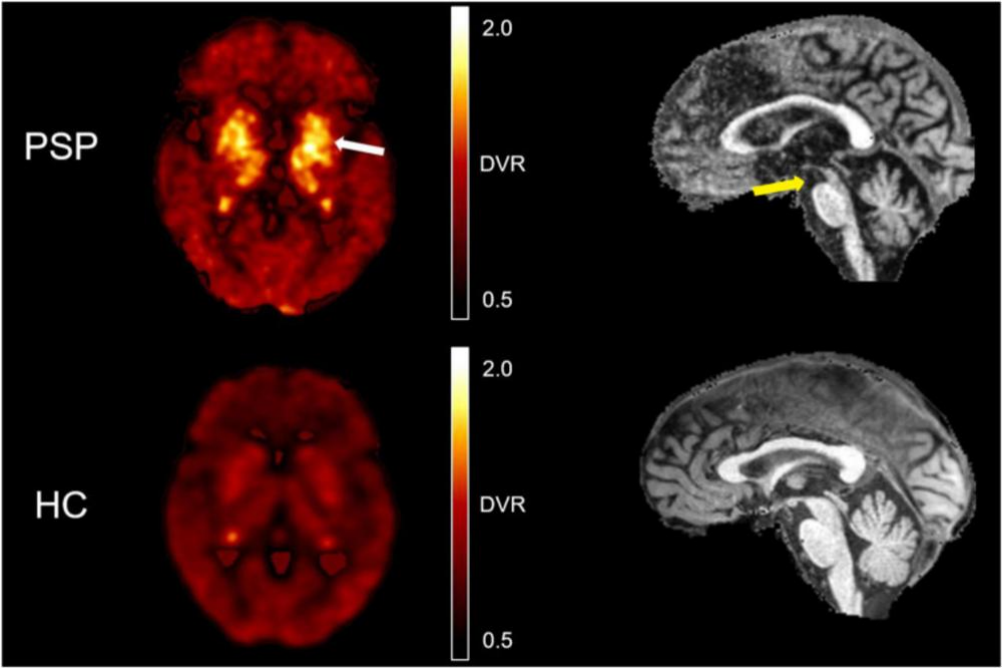
**^18^F-PI-2620 tau PET illustrating binding in the globus pallidus (white arrow on left image) and T1-weighted MRI showing midbrain atrophy (yellow arrow on right image) in PSP subjects compared with healthy controls**. Heatmap colours represent the distribution volume ratio (DVR) of the ^18^F-PI-2620. PSP, progressive supranuclear palsy; HC, healthy control; DVR, distribution volume ratio. Figure originally published in JNM. Figure from Messerschmidt *et al*.^78^

Second-generation radiotracers have improved the use of tau PET in PSP as they have higher affinity for 4R tau isoforms and reduced off-target binding.^77^^18^F-PI-2620, a second-generation tau tracer, can detect subcortical tau uptake in PSP and shows significant correlations with NfL, NfL/t-tau and GFAP levels, brain atrophy and cognitive impairment.^49^ Messerschmidt *et al.*^78^ found that using ^18^F-PI-2620 in addition to MRI measurements of midbrain atrophy improves the diagnostic accuracy of PSP compared with healthy controls. When combining visual assessment of the PET with MRI, the diagnostic sensitivity was enhanced (Fig. 2). Both sensitivity and specificity were enhanced when quantitative MRI and PET measures with the highest discriminative power were combined to result in the tau-PET/MRI-in-PSP index [MRI (midbrain/pons area ratio) × PET (globus pallidus voxel-based distribution volume ratio)]. The improvement in sensitivity was more pronounced in patients with lesser clinical severity, as measured by the PSPRS. Longitudinal studies are needed to investigate the tau-PET/MRI-in-PSP index as a biomarker of disease progression.

The development of new methods can further optimize the use of tau PET to overcome the issues of semiquantitative analysis in PSP. The lack of suitable reference regions for intensity normalization due to the diffuse spread of tau and inaccurate spatial normalization due to the small structures susceptible to tau accumulation in PSP was addressed with a normalization-free deep learning (NFDL) model for ^18^F-florzolotau PET. The NFDL successfully differentiated PSP from multiple system atrophy–Parkinsonism and healthy controls with greater accuracy than semiquantitative methods and correlated with disease severity scores on the PSPRS.^79^

### Bayesian statistical analysis methods

Considerable research has been done to determine which endpoints require the smallest sample sizes to detect a treatment effect in PSP.^11^ Also to be considered when designing and conducting clinical trials in PSP is the use of Bayesian statistical analysis methods to combat the inherent difficulties of studying a rare disease. Bayesian methods utilize prior knowledge and external information when determining the probability that a treatment has clinical value. Methods such as platform studies, use of external control data and progression modelling allow for smaller sample sizes and randomization which favours the treatment group (e.g. 2:1).^80-83^

## Future directions

Currently, the PSPRS has been accepted as the gold standard for measuring the progression of disease in PSP despite its many limitations. Therefore, objective, quantitative biomarkers able to measure disease progression are needed. Imaging or fluid-based measures would be ideal as they do not rely on subjective scoring rubrics and are not susceptible to influences such as clinician skill and practice effects.

Several biomarkers mentioned in this review have been studied longitudinally, have shown sensitivity to disease progression in PSP and do not require large sample sizes to detect treatment effect (Supplementary Table 2). These measures such as rate of midbrain atrophy should be considered for use as primary endpoints in future clinical trials. However, the FDA requires extensive research and evidence from clinical trials to prove reliability and clinical relevance of quantitative biomarkers prior to being accepted as surrogate endpoints for use in future trials. Other biomarkers which have shown diagnostic utility in PSP including QSM and second-generation tau PET tracers should be studied longitudinally to determine whether they are also sensitive to disease progression. Biomarkers studied longitudinally in other diseases such as brain-derived tau should be investigated specifically in PSP. New methods such as the use of deep learning algorithms and Bayesian approaches should also be considered to improve the feasibility of possible biomarkers.

There are many limitations when trying to identify new biomarkers. There is inconsistency in the way in which the sensitivity of potential measures has been assessed. Many studies compare the investigated biomarkers against the PSPRS as the gold standard, while others compare the biomarkers against various other measures such as cognitive assessments or brain atrophy. Some studies only measured the rate of change in the investigated biomarkers individually and did not compare them against any other measures. Approximately half of the studies in this review included participants with a possible or probable diagnosis of PSP but in many the definitiveness was not specified. Although approximately half of the studies focussed specifically on the PSP-RS subtype, others which do include all variants often combine them in one large cohort and do not investigate differences between the variants. When establishing biomarkers, it is important to determine whether they are generalizable to all PSP variants or whether they are sensitive to changes specific to individual variants only and consider this when enrolling participants into future studies and clinical trials.

## Conclusion

The PSPRS is the most frequently used endpoint to evaluate disease progression and efficacy of treatment in PSP despite not being designed for use in clinical trials. This review highlights the need for further work to establish quantitative biomarkers to measure disease progression in patients with PSP. Objective measures such as imaging or fluid-based measures are emerging as potential biomarkers; however, these require further investigation in PSP to evaluate disease-specific sensitivity and longitudinal research to validate them as reliable measures of disease progression.

## Supplementary Material

fcaf022_Supplementary_Data

## Data Availability

Data sharing is not applicable to this article as no new data were created or analysed in this study.
