# Supplementary material for: Biomarkers of disease progression in progressive supranuclear palsy for use in clinical trials
Source: Brain Commun. 2025 Jan 16;7(1):fcaf022. doi: 10.1093/braincomms/fcaf022 (PMC11775610; doi:10.1093/braincomms/fcaf022)
Supplement: fcaf022_Supplementary_Data [file fcaf022_supplementary_data.pdf]

Supplementary Table 1. Measures of longitudinal disease progression in PSP

Measure	Longitudinal change	Papers supporting	Papers opposing	Primary endpoint in trials	Secondary endpoint in trials
**Clinical**					
PSPRS	Progression (range: 7.2 – 15.8 points)	Golbe 2007[1]<br>Litvan 2014[2]<br>Bang 2016[3]<br>Street 2021[4]<br>Pereira 2022[5]<br>Grötsch 2021[6]<br>Street 2023[7]<br>Höglinger 2017[8]<br>Ghosh 2013[9]<br>Tsai 2016[10]<br>Josephs 2013[11]<br>Whitwell 2012[12]<br>Quattrone 2024[13]		NCT03068468<br>NCT05819658<br>NCT00385710<br>NCT00382824<br>NCT02985879<br>NCT01187888<br>NCT01110720<br>NCT01049399<br>NCT03391765<br>NCT04184063<br>NCT06122662<br>NCT04937530<br>NCT06162013<br>NCT06355531<br>2013-003740-23	NCT04993768<br>NCT05819658<br>NCT02460731<br>NCT02422485<br>NCT00328874<br>NCT02839642<br>NCT00703677<br>NCT01056965<br>ACTRN12620001254987
PSPRS-Ocular Motor subscore	Annual progression (range: 1.7 – 2.8 points)	Josephs 2013[11]<br>Litvan 2014[2]<br>Pereira 2022[5]<br>Whitwell 2012[12]<br>Quattrone 2024[13]			NCT05819658<br>NCT06355531
PSPRS-Gait/Midline subscore	Annual progression (range: 2.6 – 6.0 points)	Brittain 2019[14]<br>Josephs 2013[11]<br>Litvan 2014[2]<br>Ghosh 2013[9]<br>Pereira 2022[5]<br>Whitwell 2012[12]<br>Quattrone 2024[13]			NCT05819658<br>NCT06355531
PSPRS-mentation subscore	Annual progression (range: 0.6 - 1.9 points)	Litvan 2014[2]<br>Quattrone 2024[13]	Pereira 2022[5]<br>Whitwell 2012[12]		NCT05819658<br>NCT06355531

MDS-UPDRS motor section (Part III)	Annual progression (range: 7.65 – 11.3 points)	Litvan 2014[2]<br>Ghosh 2013[9]<br>Pereira 2022[5]<br>Street 2023[7]<br>Fiorenzato 2019[15]		NCT00382824	NCT03068468<br>NCT02985879<br>NCT00328874<br>NCT03391765<br>NCT00703677<br>NCT00211224<br>NCT01056965<br>NCT06122662<br>NCT06162013
CGI-C	Annual progression (range: 0.9 – 5 points)	Bang 2016[3]<br>Höglinger 2017[8]			NCT03068468<br>NCT02985879<br>NCT01110720<br>NCT01049399<br>NCT03391765<br>NCT02839642<br>NCT01056965<br>NCT06355531<br>ACTRN12620001254987<br>2013-003740-23
SEADL	Annual progression (range: -11.1 – -17.1 points)	Litvan 2014[2]<br>Street 2023[7]<br>Höglinger 2017[8]<br>Quattrone 2024[13]		NCT01110720	NCT03068468<br>NCT05819658<br>NCT00382824<br>NCT02985879<br>NCT00328874<br>NCT01049399<br>NCT03391765<br>NCT01056965<br>NCT06355531<br>NCT06162013<br>2013-003740-23
GDS					NCT00703677<br>NCT01056965<br>2013-003740-23

NNIPPS-PPS	Annual progression (25.8 points)	Payan 2011[16]			
Saccadic latency	Annual decline	Ghosh 2013[9]			NCT01056965
PSP-CDS	Annual progression (39.5%)	Piot 2020[17]			NCT06355531
Modified PSPRS	Annual progression (range: 3.2 – 4.8 points)	Grötsch 2021[6]<br>Street 2023[7]			NCT06122662
PSP QoL	Annual progression (range: 7.91 – 23.3 points)	Street 2023[7]<br>Jensen 2024[18]		NCT04184063	NCT03068468<br>NCT02985879<br>NCT01049399<br>NCT02839642<br>NCT00703677<br>NCT06355531<br>NCT06162013<br>2013-003740-23
PSP ShoQoL	Annual progression (2.46 points)	Jensen 2024[18]			
**Cognitive**					
RBANS	Annual progression (range: -5.8 – -22 points)	Bang 2016[3]<br>Duff 2020[19]	Fiorenzato 2019[15]		NCT03068468<br>NCT01056965<br>2013-003740-23
Colour trails test	Annual progression		Fiorenzato 2019[15]		NCT03068468
Phonemic fluency	Annual progression		Fiorenzato 2019[15]		NCT03068468<br>NCT01049399

FAB	Annual progression (-0.6 points)	Josephs 2013[11]<br>Litvan 2014[2]	Ghosh 2013[9]	NCT04184063	NCT05819658<br>NCT00328874<br>NCT01049399<br>NCT00703677
MMSE	Annual progression (range: -0.2 – -2.1 points)	Josephs 2013[11]<br>Litvan 2014[2]<br>Street 2021[4]	Pereira 2022[5]<br>Fiorenzato 2019[15]		NCT00382824<br>NCT00328874<br>2013-003740-23
Semantic fluency	Annual progression (-0.21)	Fiorenzato 2019[15]			
ACE-R	Annual progression (-5.3 points)	Street 2021[4]	Ghosh 2013[9]		
Hayling test	Annual progression		Ghosh 2013[9]		
MoCA	Annual progression (range: -0.8 – -2.0 points)	Street 2023[7]<br>Fiorenzato 2019[15]<br>Pavone 2023[20]	Pereira 2022[5]		NCT03068468<br>NCT05819658<br>NCT06355531<br>NCT06162013
Digit Span Sequencing	Annual progression (-0.61)	Fiorenzato 2019[15]			
Benton's JLO test	Annual progression (-0.56)	Fiorenzato 2019[15]			
**Biofluids**					
Plasma NfL					NCT04993768
CSF NfL	Annual increase	Bäckström 2015[21]<br>Magdalinou 2015[22]<br>Boxer 2014[23]	Constantinescu 2010[24]		NCT04993768<br>NCT04734379<br>NCT04253132
CSF p-tau181	Annual change		Boxer 2014[23]<br>Bäckström 2015[21]		NCT00703677<br>NCT04734379<br>NCT01056965<br>NCT04253132
CSF tau	Annual change		Boxer 2014[23]<br>Bäckström 2015[21]		NCT00703677<br>NCT01056965<br>NCT02460094<br>NCT04253132<br>2013-003740-23

Imaging					
[18F]flortaucipir PET	Annual SUVR increase	Whitwell 2019[25]			
Midbrain volume/area	Annual atrophy (range: 1.0 – 13.08%)	Whitwell 2012[12]<br>Tsai 2016[10]<br>Whitwell 2019[25]<br>Josephs 2013[11]<br>Street 2023[7]<br>Agosta 2018[26]<br>Dutt 2016[27]<br>Paviour 2006[28]<br>Paviour 2007[29]<br>Höglinger 2017[8]<br>Bang 2016[3]<br>Quattrone 2024[13]			NCT03068468<br>NCT02985879<br>2013-003740-23
Whole brain volume	Annual atrophy (range: 0.8 – 2.0%)	Whitwell 2012[12]<br>Tsai 2016[10]<br>Paviour 2006[28]<br>Paviour 2007[29]<br>Guevara 2016[30]<br>Höglinger 2017[8]<br>Bang 2016[3]<br>Josephs 2013[11]<br>Quattrone 2024[13]			NCT03068468<br>NCT02985879<br>NCT04734379
Ventricular volume	Annual enlargement (range: 6.1 – 11.8%)	Bang 2016[3]<br>Street 2023[7]<br>Paviour 2007[29]<br>Höglinger 2017[8]<br>Josephs 2013[11]			NCT03068468<br>NCT02985879<br>NCT01110720<br>NCT04734379<br>NCT01056965<br>2013-003740-23
Superior cerebellar peduncle volume	Annual atrophy (range: 2.8 – 6.9%)	Tsai 2016[10]<br>Paviour 2006[28]<br>Paviour 2007[29]<br>Quattrone 2024[13]			NCT03068468<br>NCT02985879<br>NCT04734379

Frontal lobe volume	Annual atrophy rate (range: 1.84 – 4.0%)	Josephs 2013[11]<br>Street 2023[7]<br>Höglinger 2017[8]<br>Quattrone 2024[13]			NCT03068468<br>NCT02985879<br>NCT04734379<br>2013-003740-23
Pons volume	Annual atrophy (range: 1.5 – 2.27%)	Dutt 2016[27]<br>Paviour 2006[28]<br>Paviour 2007[29]			NCT03068468<br>NCT04734379
cortical thickness	Annual change		Agosta 2018[26]		
DTI	Annual change in mean diffusivity, radial diffusivity and fractional anisotropy	Agosta 2018[26]<br>Zhang 2016[31]			NCT04734379<br>ACTRN12620001254987
ADC	Annual change	Reginold 2013[32]			
NODDI	Annual changes in isotropic volume and intracellular volume	Mitchell 2022[33]			
Free Water diffusion	Annual change in free water volume and free water corrected fractional anisotropy	Mitchell 2022[33]			
FBA	Annual decrease in fibre density	Mitchell 2022[33]			
Frontal lobe + midbrain - third ventricle volume	Annual atrophy (range: 10.38 – 12.9%)	Höglinger 2017[8]<br>Quattrone 2024[13]		ACTRN12620001254987	
MRPI 2.0	Annual change (range: 15.0 – 17.69%)	Quattrone 2020[34]<br>Quattrone 2024[13]			

PSPRS = PSP rating scale; MDS-UPDRS = Movement Disorders Society Unified Parkinson’s Disease rating scale; CGI-C = Clinical Global Impression of Change; SEADL = Schwab and England activities of daily living scale; GDS = Geriatric Depression Scale; NNIPPS-PPS = Natural History and Neuroprotection in Parkinson Plus Syndromes; PSP-CDS = Progressive Supranuclear Palsy Clinical Deficits Scale; PSP QoL = PSP Quality of life; PSP ShoQoL= PSP Short Quality of Life; RBANS = Repeatable Battery for Assessing Neuropsychological Status; FAB = Frontal Assessment Battery; MMSE = Mini Mental State Examination; ACE-R = Revised Addenbrooke's Cognitive Examination;

MoCA = Montreal Cognitive Assessment; JLO = Judgment of line orientation; NfL = Neurofilament light chain; CSF = Cerebrosoinal fluid; DTI = diffusion tensor imaging; ADC = Apparent diffusion coefficient; NODDI = Neurite Orientation Dispersion Density Imaging; FBA = Fixel Based Analysis; MPRI = Magnetic resonance parkinsonism index; NCT= National clinical trial number; ACTRN = Australian and New Zealand clinical trial registration number

Supplementary Table 2. Sample sizes required for a two-arm, 1-year follow-up therapeutic trial to detect 20%, 25%, 30%, 40% and 50% change

Measure	Paper	20% change			25% change			30% change			40% change			50% change		
		Effect size	Sample size	26% attrition	Effect size	Sample size	26% attrition	Effect size	Sample size	26% attrition	Effect size	Sample size	26% attrition	Effect size	Sample size	26% attrition
**Clinical**																
PSPRS	Stamelou 2016[17]	0.226	309	407	0.282	198	261	0.339	138	182	0.452	78	103	0.565	51	67
	Litvan 2014[2]				0.24	264	347							0.49	67	88
	Höglinger 2017[8]													1.05	58	76
	**Ghosh 2013[9]**				**1.2**	**176**	**232**							**1.2**	**45**	**59**
	**Bang 2016[3]**					**228***	**300***								**58***	**76***
	Grötsch 2021[6]	0.26	230	303	0.33	148	195	0.39	103	136	0.52	59	78	0.66	38	50
	Whitwell 2019[25]		165	217												
	**Dutt 2016[27]**					**152***	**200***								**41***	**54***
	**Tsai 2016[10]**					**142***	**187***								**38***	**50***
	**Payan 2011[16]**								**274**	**361**		**155**	**204**		**100**	**132**
	**Street 2023[7]**	**0.299**	**300**	**395**	**0.286**	**193**	**254**	**0.343**	**134**	**176**	**0.458**	**76**	**100**	**0.572**	**49**	**64**
	Piot 2020[17]							1.15	133	175				1.15	49	64
	**Pavone 2023[20]**					**123**	**162**									
	**Quattrone 2020[34]**		**383**	**504**					**171**	**225**		**97**	**128**			
	**Quattrone 2024[13]**	**1.00**	**390**	**513**				**1.00**	**174**	**229**				**1.00**	**63**	**83**
PSPRS-Ocular Motor subscore	Stamelou 2016[35]	0.153	671	883	0.191	430	566	0.126	990	1303	0.306	169	222	0.383	109	143
	Litvan 2014[2]				0.41	94	124							0.82	25	33
	Höglinger 2017[8]													0.69	133	175
	Grötsch 2021[6]	0.16	621	817	0.2	398	524	0.24	277	364	0.32	156	205	0.4	101	133
	**Pavone 2023[20]**					**341**	**449**									
	**Quattrone 2024[13]**	**0.48**	**1675**	**2204**				**0.48**	**745**	**980**				**0.48**	**269**	**354**
PSPRS-Gait/Midline subscore	Stamelou 2016[35]	0.02	384	505	0.253	246	324	0.304	172	226	0.405	97	128	0.506	63	83
	Litvan 2014[2]				0.2	382	503							0.41	97	128

	Höglinger 2017[8]													0.96	70	92
	Grötsch 2021[6]	0.24	270	355	0.3	173	228	0.36	121	159	0.48	69	91	0.61	44	58
	**Pavone 2023[20]**					**184**	**242**									
	**Quattrone 2024[13]**	**0.60**	**1076**	**1416**				**0.60**	**479**	**630**				**0.60**	**173**	**228**
PSPRS-mentation subscore	Grötsch 2021[6]	0.09	1803	2372	0.12	1154	1518	0.14	802	1055	0.19	452	595	0.23	290	382
	Litvan 2014[2]				0.23	305	401							0.45	77	101
	Stamelou 2016[35]	0.084	2226	2929	0.105	1425	1875	0.126	990	1303	0.168	558	734	0.21	357	470
	Höglinger 2017[8]													0.31	826	1087
	**Quattrone 2024[13]**	**0.44**	**2069**	**2722**				**0.44**	**920**	**1224**				**0.44**	**332**	**437**
PSPRS-10	Gewily 2024[36]		316	416					131	172					43	57
MDS-UPDRS motor section (Part III)	Litvan 2014[2]				0.17	520	684							0.35	131	172
	Guevara 2016[30]		168*	221*											27*	36*
	**Ghosh 2013[9]**				**0.73**	**486**	**639**							**0.73**	**119**	**157**
	**Payan 2011[16]**								**209**	**275**		**118**	**155**		**76**	**100**
	**Street 2023[7]**	**0.12**	**1091**	**1436**	**0.15**	**699**	**920**	**0.18**	**485**	**638**	**0.24**	**273**	**359**	**0.3**	**175**	**230**
	Piot 2020[17]							0.79	278	366				79	101	133
	**Pavone 2023[20]**					**136**	**179**									
CGI-C	Stamelou 2016[35]	0.178	498	655	0.222	319	420	0.267	222	292	0.356	126	166	0.445	81	107
	Höglinger 2017[8]													0.97	69	91
	Guevara 2016[30]		4889*	6433*											1048*	1379*
SEADL	Stamelou 2016[35]	0.191	430	566	0.239	276	363	0.287	192	253	0.383	109	143	0.478	70	92
	Litvan 2014[2]				0.2	396	521							0.4	100	132
	Höglinger 2017[8]													-0.94	73	96
	**Street 2023[7]**	**0.116**	**1168**	**1537**	**0.145**	**748**	**984**	**0.174**	**519**	**683**	**0.232**	**293**	**386**	**0.29**	**188**	**247**
	**Dutt 2016[27]**		**86***	**113***											**91***	**120***
	**Tsai 2016[10]**					**184***	**242***								**48***	**63***
	**Payan 2011[16]**								**179**	**236**		**102**	**134**		**65**	**86**
	Piot 2020[17]							-0.74	319	420				-0.74	116	153
GDS	Stamelou 2016[35]	0.033	13989	18407	0.042	8954	11782	0.05	6218	8182	0.067	3498	4603	0.084	2240	2947

NNIPPS-PPS	**Payan 2011[16]**								**112**	**147**		**63**	**83**		**40**	**53**
PSP-CDS	Piot 2020[17]							1	177	233				1	65	86
modified PSPRS	Grötsch 2021[6]	0.25	248	326	0.32	159	209	0.38	111	146	0.51	63	83	0.63	41	54
	**Street 2023[7]**	**0.228**	**303**	**399**	**0.286**	**193**	**254**	**0.343**	**134**	**176**	**0.457**	**76**	**100**	**0.571**	**49**	**64**
	Piot 2020[17]							0.49	742	976				0.49	268	353
PSP QoL	**Street 2023[7]**	**0.176**	**508**	**668**	**0.22**	**325**	**428**	**0.265**	**224**	**295**	**0.353**	**127**	**167**	**0.441**	**82**	**108**
**Cognitive**																
RBANS	**Bang 2016[3]**					**304***	**400***								**77***	**101***
	**Tsai 2016[10]**					**249***	**328***								**65***	**86***
Colour trails test	**Bang 2016[3]**					**193***	**254***								**49***	**64***
Phonemic fluency	**Bang 2016[3]**					**275***	**362***								**70***	**92***
FAB	Stamelou 2016[35]													0.112	1244	1637
	**Ghosh 2013[9]**				**0.01**	**2511637**	**3304786**							**0.01**	**2511637**	**3304786**
	Guevara 2016[30]		22688*	29853*											3630*	4776*
MMSE	Litvan 2014[2]				0.32	151	199							0.65	39	51
ACE-R	**Ghosh 2013[9]**				**0.27**	**3447**	**4536**							**0.27**	**863**	**1136**
Hayling test	**Ghosh 2013[9]**				**0.02**	**627910**	**826197**							**0.02**	**627910**	**826197**
MoCA	**Street 2023[7]**	**0.055**	**5190**	**6829**	**0.069**	**3298**	**4339**	**0.082**	**2336**	**3074**	**0.11**	**1298**	**1708**	**0.137**	**837**	**1101**
	Piot 2020[17]							-0.31	1853	2438			0	-0.31	668	879
	**Pavone 2023[20]**					**851**	**1120**									
**Imaging**																
[18F]AV1451 PET	Whitwell 2019[25]		741	975												
Midbrain volume/area	**Street 2023[7]**	**0.023**	**29675**	**39046**	**0.029**	**18667**	**24562**	**0.035**	**12815**	**16862**	**0.047**	**7107**	**9351**	**0.059**	**4511**	**5936**
	Höglinger 2017[8]	-1.33	223	293				-1.33	100	132				-1.33	37	49
	**Bang 2016[3]**					**219***	**288***								**55***	**72***
	Paviour 2007[29]		330*	434*					842*	1108*		82*	108*			
	Whitwell 2019[25]		94	124												
	**Dutt 2016[27]**		**77***	**101***											**23***	**30***

	**Tsai 2016[10]**					**139***	**183***								**36***	**47***
	**Quattrone 2024[13]**	**-1.43**	**192**	**253**				**-1.43**	**86**	**113**				**-1.43**	**31**	**41**
Whole brain volume	Höglinger 2017[8]	-1.1	325	428				-1.1	145	191				-1.1	53	70
	**Bang 2016[3]**					**229***	**301***								**58***	**76***
	Paviour 2007[29]		898	1182					399	525		225	296			
	**Tsai 2016[10]**					**358***	**471***								**87***	**114***
	Guevara 2016[30]		83*	109*											14*	18*
	**Quattrone 2024[13]**	**-0.94**	**448**	**589**				**-0.94**	**200**	**263**				**-0.94**	**72**	**95**
Ventricular volume	**Street 2023[7]**	**0.341**	**136**	**179**	**0.426**	**87**	**114**	**0.511**	**61**	**80**	**0.682**	**35**	**46**	**0.852**	**23**	**30**
	Höglinger 2017[8]	1.42	195	257				1.42	88	116				1.42	32	42
	Paviour 2007[29]		1213*	1596*					539*	709*		303*	399*			
	**Tsai 2016[10]**					**130***	**171***								**35***	**46***
Superior Cerebellar Peduncle atrophy	Höglinger 2017[8]	-0.89	493	649				-0.89	220	289				-0.89	80	105
	**Bang 2016[3]**					**378***	**497***								**96***	**126***
	Paviour 2007[29]		682*	897*					303*	399*		170*	224*			
	**Tsai 2016[10]**					**417***	**549***								**108***	**142***
	**Quattrone 2024[13]**	**-1.17**	**286**	**376**				**-1.17**	**128**	**168**				**-1.17**	**46**	**61**
Frontal lobe volume	**Street 2023[7]**	**0.267**	**221**	**291**	**0.334**	**142**	**187**	**0.401**	**99**	**130**	**0.534**	**56**	**74**	**0.668**	**36**	**47**
	Höglinger 2017[8]	-1.22	263	346				-1.22	118	155				-1.22	43	57
	**Quattrone 2024[13]**	**-0.72**	**764**	**1005**				**-0.72**	**340**	**447**				**-0.72**	**123**	**162**
Frontal lobe + midbrain - third ventricle volume	Höglinger 2017[8]	-1.83	119	157				-1.83	54	71				-1.83	20	26
	**Quattrone 2024[13]**	**-1.34**	**220**	**289**				**-1.34**	**98**	**129**				**-1.34**	**36**	**47**
Pons volume	**Street 2023[7]**	**0.186**	**455**	**599**	**0.232**	**293**	**386**	**0.278**	**204**	**268**	**0.371**	**115**	**151**	**0.464**	**74**	**97**
	Höglinger 2017[8]	-1.12	312	411				-1.12	139	183			0	-1.12	51	67
	Paviour 2007[29]		499*	657*					221*	291*		125*	164*			
	**Dutt 2016[27]**		**86***	**113***											**25***	**33***
	**Quattrone 2024[13]**	**-1.27**	**244**	**321**				**-1.27**	**109**	**143**				**-1.27**	**40**	**53**
Brainstem volume	**Quattrone 2024[13]**	**-1.44**	**190**	**250**				**-1.44**	**85**	**112**				**-1.44**	**31**	**41**
MRPI 2.0	**Quattrone 2020[34]**		**213**	**280**					**96**	**126**		**54**	**71**			

	**Quattrone 2024[13]**	**0.69**	**832**	**1095**				**0.69**	**270**	**355**				**0.69**	**134**	**176**

Results are from t-tests with 5% significance and 80% power, * indicates power of 90%

Papers in bold font are based on only PSP-Richardson's Syndrome subjects.

PSPRS = PSP rating scale; MDS-UPDRS = Movement Disorders Society Unified Parkinson's Disease rating scale; CGI-C = Clinical Global Impression of Change; SEADL = Schwab and England activities of daily living scale; GDS = Geriatric Depression Scale; NNIPPS-PPS = Natural History and Neuroprotection in Parkinson Plus Syndromes; PSP-CDS = Progressive Supranuclear Palsy Clinical Deficits Scale; PSP QoL = PSP Quality of life; RBANS = Repeatable Battery for Assessing Neuropsychological Status; FAB = Frontal Assessment Battery; MMSE = Mini Mental State Examination; ACE-R = Revised Addenbrooke's Cognitive Examination; MoCA = Montreal Cognitive Assessment; MPRI = Magnetic resonance parkinsonism index

REFERENCES

1. Golbe LI, Ohman-Strickland PA. A clinical rating scale for progressive supranuclear palsy. *Brain*. 2007;130(6):1552-1565. doi:10.1093/brain/awm032

2. Litvan I, Kong M. Rate of decline in progressive supranuclear palsy. *Movement Disorders*. 2014;29(4):463-468. doi:10.1002/mds.25843

3. Bang J, Lobach IV, Lang AE, et al. Predicting Disease Progression in Progressive Supranuclear Palsy in Multicenter Clinical Trials. *Parkinsonism Relat Disord*. 2016;28:41-48. doi:10.1016/j.parkreldis.2016.04.014

4. Street D, Malpetti M, Rittman T, et al. Clinical progression of progressive supranuclear palsy: impact of trials bias and phenotype variants. *Brain Commun*. 2021;3(3):fcab206. doi:10.1093/braincomms/fcab206

5. Pereira MF, Buchanan T, Höglinger GU, et al. Longitudinal changes of early motor and cognitive symptoms in progressive supranuclear palsy: the OxQUIP study. *BMJ Neurol Open*. 2022;4(1):e000214. doi:10.1136/bmjno-2021-000214

6. Grötsch MT, Respondek G, Colosimo C, et al. A Modified Progressive Supranuclear Palsy Rating Scale. *Movement Disorders*. 2021;36(5):1203-1215. doi:10.1002/mds.28470

7. Street D, Jabbari E, Costantini A, et al. Progression of atypical parkinsonian syndromes: PROSPECT-M-UK study implications for clinical trials. *Brain*. Published online March 28, 2023:awad105. doi:10.1093/brain/awad105

8. Höglinger GU, Schöpe J, Stamelou M, et al. Longitudinal magnetic resonance imaging in progressive supranuclear palsy: A new combined score for clinical trials. *Mov Disord*. 2017;32(6):842-852. doi:10.1002/mds.26973

9. Ghosh BCP, Carpenter RHS, Rowe JB. A Longitudinal Study of Motor, Oculomotor and Cognitive Function in Progressive Supranuclear Palsy. *PLoS One*. 2013;8(9):e74486. doi:10.1371/journal.pone.0074486

10. Tsai RM, Lobach I, Bang J, et al. Clinical correlates of longitudinal brain atrophy in progressive supranuclear palsy. *Parkinsonism Relat Disord*. 2016;28:29-35. doi:10.1016/j.parkreldis.2016.04.006

11. Josephs KA, Xia R, Mandrekar J, et al. Modeling trajectories of regional volume loss in progressive supranuclear palsy. *Movement Disorders*. 2013;28(8):1117-1124. doi:10.1002/mds.25437

12. Whitwell JL, Xu J, Mandrekar J, Gunter JL, Jack Jr. CR, Josephs KA. Imaging measures predict progression in progressive supranuclear palsy. *Movement Disorders*. 2012;27(14):1801-1804. doi:10.1002/mds.24970

13. Quattrone A, Franzmeier N, Huppertz HJ, et al. Magnetic Resonance Imaging Measures to Track Atrophy Progression in Progressive Supranuclear Palsy in Clinical Trials. *Movement Disorders*. 2024;39(8):1329-1342. doi:10.1002/mds.29866

14. Brittain C, McCarthy A, Irizarry MC, et al. Severity dependent distribution of impairments in PSP and CBS: Interactive Visualizations. *Parkinsonism Relat Disord*. 2019;60:138-145. doi:10.1016/j.parkreldis.2018.08.025

15. Fiorenzato E, Antonini A, Camparini V, Weis L, Semenza C, Biundo R. Characteristics and progression of cognitive deficits in progressive supranuclear palsy vs. multiple system atrophy and Parkinson's disease. *J Neural Transm*. 2019;126(11):1437-1445. doi:10.1007/s00702-019-02065-1

16. Payan CAM, Viallet F, Landwehrmeyer BG, et al. Disease Severity and Progression in Progressive Supranuclear Palsy and Multiple System Atrophy: Validation of the NNIPPS – PARKINSON PLUS SCALE. *PLoS One*. 2011;6(8):e22293. doi:10.1371/journal.pone.0022293

17. Piot I, Schweyer K, Respondek G, et al. The Progressive Supranuclear Palsy Clinical Deficits Scale. *Movement Disorders*. 2020;35(4):650-661. doi:10.1002/mds.27964

18. Jensen I, Stiel S, Bebermeier S, et al. A Short Progressive Supranuclear Palsy Quality of Life Scale. *Movement Disorders*. 2024;39(9):1602-1609. doi:10.1002/mds.29936

19. Duff K, Randolph C, Boxer AL. Cognitive decline on the Repeatable Battery for the Assessment of Neuropsychological Status in Progressive Supranuclear Palsy. *Clin Neuropsychol*. 2020;34(3):529-540. doi:10.1080/13854046.2019.1670865

20. Pavone C, Weigand SW, Ali F, et al. Longitudinal clinical decline and baseline predictors in progressive supranuclear palsy. *Parkinsonism & Related Disorders*. 2023;107:105290. doi:10.1016/j.parkreldis.2023.105290

21. Bäckström DC, Eriksson Domellöf M, Linder J, et al. Cerebrospinal Fluid Patterns and the Risk of Future Dementia in Early, Incident Parkinson Disease. *JAMA Neurology*. 2015;72(10):1175-1182. doi:10.1001/jamaneurol.2015.1449

22. Magdalinou NK, Paterson RW, Schott JM, et al. A panel of nine cerebrospinal fluid biomarkers may identify patients with atypical parkinsonian syndromes. *J Neurol Neurosurg Psychiatry*. 2015;86(11):1240-1247. doi:10.1136/jnnp-2014-309562

23. Boxer AL, Lang AE, Grossman M, et al. Davunetide for Progressive Supranuclear Palsy: a multicenter, randomized, double-blind, placebo controlled trial. *Lancet Neurol*. 2014;13(7):676-685. doi:10.1016/S1474-4422(14)70088-2

24. Constantinescu R, Rosengren L, Johnels B, Zetterberg H, Holmberg B. Consecutive analyses of cerebrospinal fluid axonal and glial markers in Parkinson's disease and atypical parkinsonian disorders. *Parkinsonism & Related Disorders*. 2010;16(2):142-145. doi:10.1016/j.parkreldis.2009.07.007

25. Whitwell JL, Tosakulwong N, Schwarz CG, et al. MRI Outperforms [18F]AV-1451 PET as a Longitudinal Biomarker in Progressive Supranuclear Palsy. *Mov Disord*. 2019;34(1):105-113. doi:10.1002/mds.27546

26. Agosta F, Caso F, Ječmenica-Lukić M, et al. Tracking brain damage in progressive supranuclear palsy: a longitudinal MRI study. *J Neurol Neurosurg Psychiatry*. 2018;89(7):696-701. doi:10.1136/jnnp-2017-317443

27. Dutt S, Binney RJ, Heuer HW, et al. Progression of brain atrophy in PSP and CBS over 6 months and 1 year. *Neurology*. 2016;87(19):2016-2025. doi:10.1212/WNL.0000000000003305

28. Paviour DC, Price SL, Jahanshahi M, Lees AJ, Fox NC. Longitudinal MRI in progressive supranuclear palsy and multiple system atrophy: rates and regions of atrophy. *Brain*. 2006;129(4):1040-1049. doi:10.1093/brain/awl021

29. Paviour DC, Price SL, Lees AJ, Fox NC. MRI derived brain atrophy in PSP and MSA-P. *J Neurol*. 2007;254(4):478-481. doi:10.1007/s00415-006-0396-4

30. Guevara C, Bulatova K, Barker GJ, Gonzalez G, Crossley N, Kempton MJ. Whole-Brain Atrophy Rate in Idiopathic Parkinson's Disease, Multiple System Atrophy, and Progressive Supranuclear Palsy. *Parkinsons Dis*. 2016;2016:9631041. doi:10.1155/2016/9631041

31. Zhang Y, Walter R, Ng P, et al. Progression of Microstructural Degeneration in Progressive Supranuclear Palsy and Corticobasal Syndrome: A Longitudinal Diffusion Tensor Imaging Study. Yang S, ed. *PLoS ONE*. 2016;11(6):e0157218. doi:10.1371/journal.pone.0157218

32. Reginold W, Lang AE, Marras C, Heyn C, Alharbi M, Mikulis DJ. Longitudinal quantitative MRI in multiple system atrophy and progressive supranuclear palsy. *Parkinsonism & Related Disorders*. 2014;20(2):222-225. doi:10.1016/j.parkreldis.2013.10.002

33. Mitchell T, Wilkes BJ, Archer DB, et al. Advanced diffusion imaging to track progression in Parkinson's disease, multiple system atrophy, and progressive supranuclear palsy. *Neuroimage Clin*. 2022;34:103022. doi:10.1016/j.nicl.2022.103022

34. Quattrone A, Morelli M, Quattrone A, et al. Magnetic Resonance Parkinsonism Index for evaluating disease progression rate in progressive supranuclear palsy: A longitudinal 2-year study. *Parkinsonism & Related Disorders*. 2020;72:1-6. doi:10.1016/j.parkreldis.2020.01.019

35. Stamelou M, Schöpe J, Wagenpfeil S, et al. Power Calculations and Placebo Effect for Future Clinical Trials in Progressive Supranuclear Palsy. *Mov Disord*. 2016;31(5):742-747. doi:10.1002/mds.26580

36. Gewily M, Plan EL, Yousefi E, et al. Quantitative Comparisons of Progressive Supranuclear Palsy Rating Scale Versions Using Item Response Theory. *Movement Disorders*. n/a(n/a). doi:10.1002/mds.30001
